# Scapula alata in early breast cancer patients enrolled in a randomized clinical trial of post-surgery short-course image-guided radiotherapy

**DOI:** 10.1186/1477-7819-10-86

**Published:** 2012-05-16

**Authors:** Nele Adriaenssens, Mark De Ridder, Pierre Lievens, Hilde Van Parijs, Marian Vanhoeij, Geertje Miedema, Mia Voordeckers, Harijati Versmessen, Guy Storme, Jan Lamote, Stephanie Pauwels, Vincent Vinh-Hung

**Affiliations:** 1 Breast Clinic, Oncologic Surgery, Universitair Ziekenhuis Brussel, Laarbeeklaan 101, 1090, Brussels, Belgium; 2 Department of Radiotherapy, Oncology Centre, Universitair Ziekenhuis Brussel, Laarbeeklaan 101, 1090, Brussels, Belgium; 3 Physical Therapy Department, Vrije Universiteit Brussel, Laarbeeklaan 103, 1090, Brussels, Belgium; 4 Radiation Oncology, Geneva University Hospitals, Rue Gabrielle-Perret-Gentil 4, 1211, Geneva 14, Switzerland

**Keywords:** Breast cancer, Surgery, Radiation treatment, Complications, Winged scapula, Scapular winging, Long thoracic nerve, Multiple outcomes, Shoulder/arm morbidity, Lymphedema

## Abstract

**Background:**

Scapula alata (SA) is a known complication of breast surgery associated with palsy of the serratus anterior, but it is seldom mentioned. We evaluated the risk factors associated with SA and the relationship of SA with ipsilateral shoulder/arm morbidity in a series of patients enrolled in a trial of post-surgery radiotherapy (RT).

**Methods:**

The trial randomized women with completely resected stage I-II breast cancer to short-course image-guided RT, versus conventional RT. SA, arm volume and shoulder-arm mobility were measured prior to RT and at one to three months post-RT. Shoulder/arm morbidities were computed as a post-RT percentage change relative to pre-RT measurements.

**Results:**

Of 119 evaluable patients, 13 (= 10.9%) had pre-RT SA. Age younger than 50 years old, a body mass index less than 25 kg/m2, and axillary lymph node dissection were significant risk factors, with odds ratios of 4.8 (*P =* 0.009), 6.1 (*P* = 0.016), and 6.1 (*P* = 0.005), respectively. Randomization group was not significant. At one to three months’ post-RT, mean arm volume increased by 4.1% (*P* = 0.036) and abduction decreased by 8.6% (*P* = 0.046) among SA patients, but not among non-SA patients. SA resolved in eight, persisted in five, and appeared in one patient.

**Conclusion:**

The relationship of SA with lower body mass index suggests that SA might have been underestimated in overweight patients. Despite apparent resolution of SA in most patients, pre-RT SA portended an increased risk of shoulder/arm morbidity. We argue that SA warrants further investigation. Incidentally, the observation of SA occurring after RT in one patient represents the second case of post-RT SA reported in the literature.

## Background

Scapula alata (SA), also called scapular winging, winged scapula or alar scapula, is a condition in which the medial border and angulus inferior of the scapula protrudes prominently from the thorax [1]. It can arise from numerous pathologic processes, which lead to a deficiency of the muscles that play a role in pulling the scapula towards the thoracic wall -serratus anterior, trapezius (pars descendens), and rhomboids - such as by injury of the long thoracic nerve (the most common cause), the spinal accessory nerve and the dorsal scapular nerve respectively [2]. The condition can be distressful and debilitating [3]. Depending on the underlying causes, the compensatory muscular activity required to improve shoulder stability can be associated with secondary pain and spasm due to muscle imbalances or tendinitis around the shoulder joint [4].

SA associated with serratus anterior palsy is a known complication of breast and axillary surgery. In 1825, Velpeau cautioned that any axillary operation should be carefully carried out to avoid damaging the long thoracic nerve. He described that symptoms of damage would be a displacement of the scapula backwards and upwards and the inability of the scapula to come into close apposition with the thorax [5] (page 303). Yet despite the long-known history, SA has seldom been investigated in breast cancer research, in contrast with other domains, such as sports medicine. There are considerably large variations in the reported incidence of SA after breast surgery, ranging from 0% to 74.7% [6-10], without clear explanation of the variability.

Our institution conducted, from 2007 to 2011, the TomoBreast randomized clinical trial which compared post-operative short-course image-guided radiotherapy (IGRT) with conventional radiotherapy (conventional RT) for early breast cancer. SA was not a specified endpoint of the trial. Nevertheless, it was systematically assessed in patients enrolled in the trial. We believe that an analysis of the trial’s data might provide new insight into the clinical significance of SA. In the present study, our aims are to evaluate the incidence of SA among the patients who participated in the trial, to identify patients’ characteristics associated with SA, and to evaluate the relationship of SA with physical measurements of arm volume and shoulder-arm mobility.

## Methods

### Selection of patients

The study population consisted of women who participated in the TomoBreast clinical trial (NCT00459628, ISRCTN21164902) approved by the University Hospital of Brussels’ ethics board. The trial recruited women aged 18 years or older, presenting with a primary breast carcinoma completely removed by mastectomy or by breast-conserving surgery, pathological stage pT1-3N0M0 or pT1-2N1M0 with pathological nodal status assessed by axillary lymph node dissection (ALND) or by sentinel nodes biopsy (SNB), who were to receive post-surgery radiotherapy. Women who gave written informed consent were allocated to either a control group or to an experimental group by computer randomization. In order to reduce the risk of imbalance due to the small size of the trial, randomization used Efron’s biased coin design: instead of a fixed 1/2 probability, the probability of a new patient being allocated to the control or to the experimental group was assigned as 1/3, as 1/2, or as 2/3, depending on how many preceding patients, stratified by nodal status, type of surgery, and chemotherapy sequence, had been previously allocated in one or the other group [11]. In the control group, a dose of 50 Gy was delivered in 25 fractions over five weeks to the chest wall using tangential photon fields, and in cases of pN1 status, to the supraclavicular, infraclavicular and axillary nodes using an anterior field matched to the tangential fields. Breast-conserved patients received, in addition, a sequential boost of 16 Gy delivered in 8 fractions over two weeks to the initial tumor bed using a direct electron field. In the experimental group, a dose of 42 Gy was delivered in 15 fractions over three weeks to the chest wall in cases of mastectomy, or to the whole breast in cases of breast-conserving surgery, and to the supraclavicular, infraclavicular and axillary nodes in cases of pN1 status, using the image-guided radiotherapy system TomoTherapy (TomoTherapy Inc., Madison, WI, USA). Breast-conserved patients received a simultaneous integrated boost of 9 Gy delivered in 15 fractions over the three weeks.

Per protocol, radiotherapy had to start within six weeks of breast surgery, or, in case of adjuvant chemotherapy, within six weeks after completion of the adjuvant chemotherapy. Quality of life, arm mobility and volume, pulmonary function and heart function tests were scheduled prior to radiotherapy, at one to three months after completion of radiotherapy, then yearly. The primary endpoint of the trial was the combined pulmonary and cardiac toxicities as determined by medical imaging and functional tests during follow-up versus pre-treatment evaluation. The secondary endpoint was locoregional recurrence. Formal comparisons of the endpoints and quality of life between treatment groups are ongoing but are not the purpose of the present study. The focus of the study is scapula alata and the physical therapy assessment made prior to radiotherapy and at the first follow-up one to three months after radiotherapy.

Written informed consent was obtained from the patients for publication of this report and any accompanying images.

### Physical therapy assessment

Patient’s subjective arm symptoms, physical shoulder-arm evaluation, and presence or not of scapula alata were assessed by a physical therapist after the patient’s consent to participate in the trial but before radiotherapy (pre-RT evaluation) and at one to three months after the last radiotherapy session.

Subjective arm symptoms were recorded as present or absent. Arm symptoms were considered present when the patient reported for the operated arm/hand any symptom of dysesthesia, heaviness, swelling, fatigue, more effort needed, warmth, burning or pain. Arm symptoms were considered absent when the patient reported none of these symptoms.

**Figure 1:**
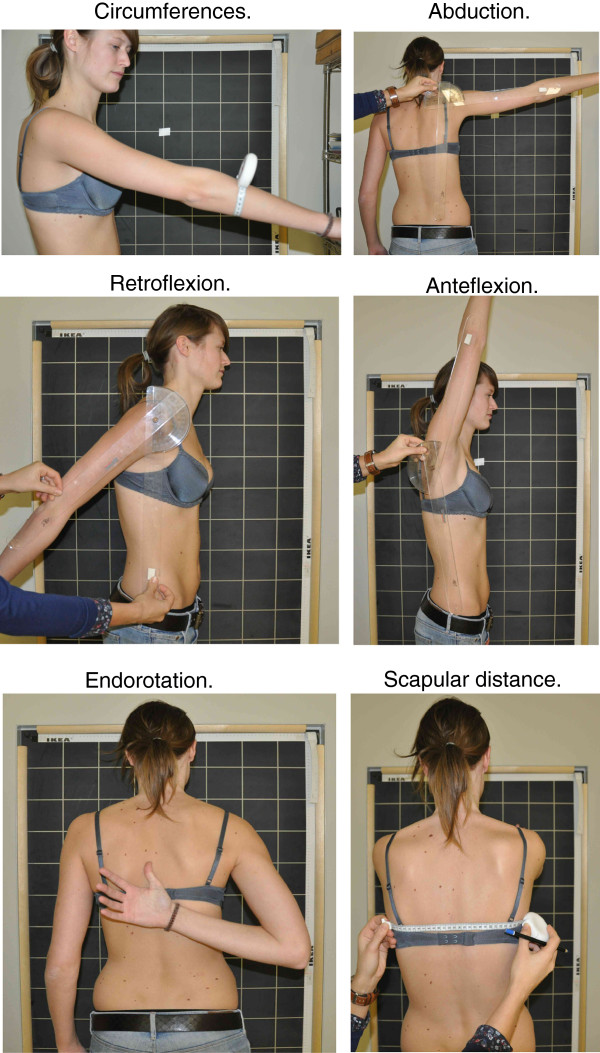
**Physical measurements**. Measured at five locations (marked with a dermographic pencil but not visible in the picture). The tape box has a push button to maintain the same tension. Abduction: running angle by lateral elevation, measured between the midline of the hemibody (goniometer’s arm in line with the ipsilateral posterior superior iliac spine), and the midline of the upper arm (goniometer’s arm in line with the lateral epicondyle of the humerus). Retroflexion: running angle by posterior elevation, measured between the midline of the body (goniometer’s arm in line downward with the trochanter major), and the midline of the upper arm (goniometer’s arm in line with the lateral epicondyle of the humerus). Anteflexion: running angle by anterior elevation, measured between the midline of the body (goniometer’s arm in line downward with the trochanter major), and the midline of the upper arm (goniometer’s arm in line with the lateral epicondyle of the humerus). Endorotation: the thumb as close as possible to C7. The number of vertebrae between C7 and the vertebra that can be reached with the thumb is marked as endorotation measurement. Scapular distance: with the patient’s arms held actively at 90° anteflexion, the distance of the scapula inferior angle to the spine, perpendicularly to the spine, is measured with a tape.

Shoulder-arm evaluation recorded the following measurements (Figure 1):

Arm volume, computed from circumferential measurements using the mean of the frustum sign and the cylinder model method as detailed in Appendix 1 of Additional file 1 [12].

Maximum range of active lateral elevation of the arm (abduction).

Maximum range of active forward elevation of the arm (anteflexion).

Maximum range of active backward elevation of the arm (retroflexion).

Maximal functional endorotation measured by counting the vertebrae between C7 and the most cranial vertebra the patient could reach with her thumb on her back.

Scapular distance (the lateral scapular slide test), measured as the distance between the spine and the angulus inferior of the scapula, with the arms elevated 90° in the scapular plane [13].

Note that impairment of arm mobility is indicated by decreased abduction, anteflexion, and/or retroflexion. However, impairment of endorotation would be marked by the inability of the hand on the back to reach closer to the neck, with consequently an increased count of vertebrae. Likewise, shoulder injury might entail decreased ability of the scapula to slide toward the spine, with consequently an increased scapular distance [14,15]. All measurements were made on both arms, ipsilaterally and contralaterally to the operated side. For the present study, we took into consideration only measurements pertaining to the ipsilateral arm.

**Figure 2:**
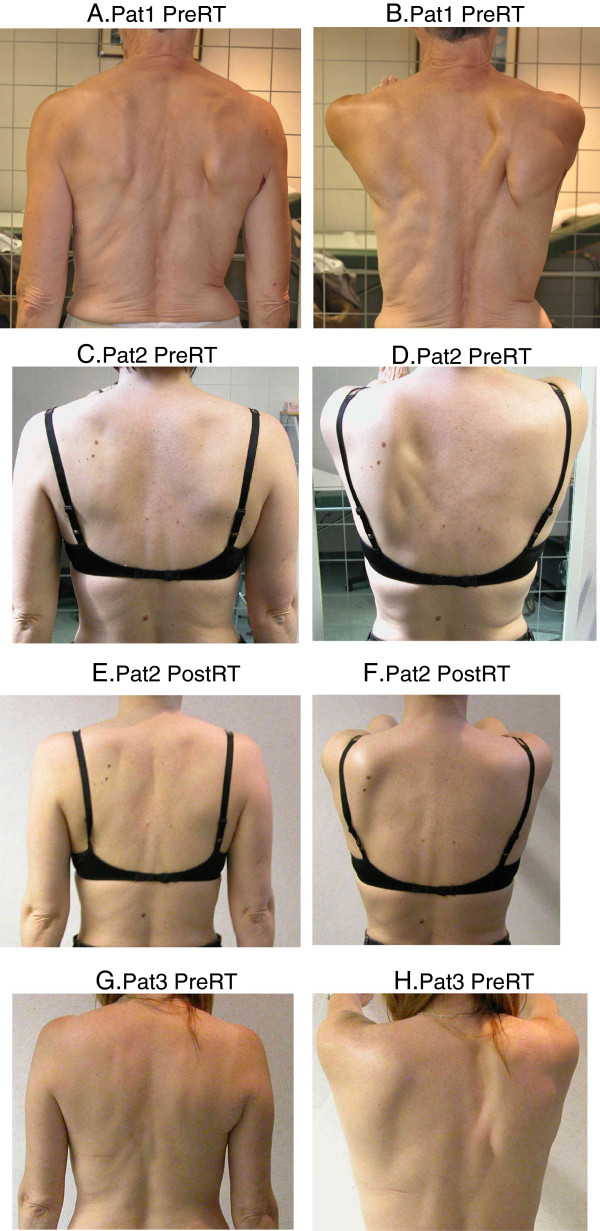
**Scapular winging.** Scapula alata assessed in TomoBreast patients. **(A)** Patient 1 pre-RT arms relaxed, **(B)** Patient 1 pre-RT arms elevated, **(C)** Patient 2 pre-RT arms relaxed, **(D)** Patient 2 pre-RT arms elevated, **(E)** Patient 2 post-RT arms relaxed, **(F)** Patient 2 post-RT arms elevated, **(G)** Patient 3 pre-RT arms relaxed and **(H)** Patient 3 pre-RT arms elevated.

Scapula alata was assessed through visual observation of tilting and winging of the scapulae (Figure 2). The observation was performed with the subject instructed to stand relaxed and perform active elevation of the arms in the scapular plane until shoulder height. No differentiation in the amount of elevation was specified. The patient was observed from dorsal (frontal plane) and lateral (sagittal plane). Normally, the inferior angle should be flat against the chest wall [16] and the scapulae should be 30° internally rotated with respect to the frontal plane [17]. Scapular positioning was deemed impaired when:

the inferior angle of the scapula became prominent dorsally (rotating about the horizontal axis - tilting;

the entire medial border of the scapula became prominent dorsally (rotation about the vertical axis - winging.

If one or both criteria listed above were fulfilled, we scored scapula alata as 1 (SA present), only if there was a clear observation of the positioning fault. If none of the criteria were met, we judged scapula alata as 0 (no SA). Each position was observed and evaluated once.

### Statistical analyses

In order to evaluate shoulder/arm morbidity on a common scale and to avoid reliance on the contralateral arm measurements, we computed the outcome of a shoulder/arm measurement as the percentage change of the measurement that occurred over time, between pre-RT assessment (= time T0) and post-RT assessment (= time T1) of the ipsilateral arm. That is, for volume, the percent change of volume was computed as: 100 x (volume of arm at T1 – volume of arm at T0)/volume of arm at T0. Likewise, for abduction, the percentage change of abduction was computed as 100 x (abduction at T1 – abduction at T0)/abduction at T0, and so on for retroflexion, anteflexion, endorotation, and scapular distance. The percentage changes of the measurements were analyzed as continuous variables, and were also analyzed as categorized variables. Categorization used cutoffs for limb edema and for motion impairment derived from the Common Terminology Criteria for Adverse Events version 4.0 (CTCAE 4.03) [18]. For arm swelling, the cutoffs applied were Grade 0 = less than 5%, Grade 1 = 5% to less than 10%, Grade 2 = 10% to less than 30%, and Grade 3 = 30% or more increase of arm volume, where percentage increases are computed as defined above, in order to avoid reliance on the contralateral limb [19]. For loss of range of motion, the cutoffs applied were Grade 0 = 5% or less, Grade 1 = more than 5% to 25%, Grade 2 = more than 25% to 50%, and Grade 3 = more than 50% loss of motion. CTCAE 4.03 does not specify a lower bound in the definition of Grade 1 toxicity. We implemented a lower bound of 5% in order to take into account the normal variability of range of motion [20-25].

Fisher’s exact test was used for the analysis of data categorized in contingency tables [26]. Cochran-Armitage’s trend test was used for ordinal tables [27] (pp 504-509). Odds ratio relating SA with patients’ characteristics were computed by conditional maximum likelihood. Logistic regression was used to evaluate the multivariate association of patients’ characteristics with SA. Significance testing of continuous measurements used Student’s t-test. *P*-values from one-sided or two-sided tests are indicated as 1*P* or 2*P*, respectively. The overall assessment of multiple outcomes used Brown’s method to combine non-independent tests of significance [28].

All statistical computations used R version 2.14.1 [29]. Missing data were imputed using the method of multivariate imputation by chained equations from package ‘mice’ [30]. Variables used for imputation are listed in Appendix 2 of Additional file 1. Fisher’s exact test and odds ratios were computed using the function ‘fisher.test’. Ordinal test of proportions used the function ‘prop.trend.test’. Logistic regression used the function ‘glm’ [31]. Brown’s method for combining non-independent tests of significance [28] was computed using an in-house R script (Additional file 2).

## Results

The TomoBreast trial was opened to accrual on 1 May 2007, and closed accrual on 31 August 2011. A total of 123 women consented to participate, two of whom were ineligible, one presented bilateral breast cancer, the other retracted participation. Of the 121 eligible patients, two had no follow-up physical examination, leaving 119 patients available for analysis. Missing data were pre-RT arms symptoms not recorded in eleven patients, and the side of the dominant arm not recorded in one patient (Table 1). One patient was in a wheelchair at the pre-RT assessment, so retroflexion of both arms could not be measured and were assigned as missing. One patient had long-standing contralateral arm paralysis, but this did not affect the present analyses, which did not rely on contralateral arm measurements.

**Table 1:** Patients’ characteristics

Characteristic (mean)	n	Scapula alata		Odds ratio	2*P*
		Not present	Present		
		n = 106	n = 13		
		n (row%)	n (row%)			
Age (mean 56.6)				4.8	0.009
<50 years	34	26 (76.5)	8 (23.5)		
> = 50	85	80 (94.1)	5 (5.9)		
Weight pre-RT (mean 68.4)				4.3	0.071
<70 kg	70	59 (84.3)	11 (15.7)		
> = 70	49	47 (95.9)	2 (4.1)		
Height (mean 1.62)				1.7	0.346
<1.60 m	33	28 (84.8)	5 (15.2)		
> = 1.60	86	78 (90.7)	8 (9.3)		
Body mass index pre-RT (mean 25.9)				6.1	0.016
<25 kg/m2	61	50 (82)	11 (18)		
> = 25	58	56 (96.6)	2 (3.4)		
Arm symptoms pre-RT				0.8	0.726
No	86	76 (88.4)	10 (11.6)		
Yes	22	19 (86.4)	3 (13.6)		
Missing	11	11 (100)	0 (0)		
Side of surgery is dominant arm				0.5	0.378
No	63	58 (92.1)	5 (7.9)		
Yes	55	47 (85.5)	8 (14.5)		
Missing	1	1 (100)	0 (0)		
Breast surgery				2.3	0.221
Mastectomy	43	36 (83.7)	7 (16.3)		
Breast-conserving	76	70 (92.1)	6 (7.9)		
Axillary surgery				6.1	0.005
Axillary dissection	47	37 (78.7)	10 (21.3)		
Sentinel node	72	69 (95.8)	3 (4.2)		
Number of nodes examined (mean 8.2)				0.3	0.133
<10	77	72 (93.5)	5 (6.5)		
> = 10	42	34 (81)	8 (19)		
Chemotherapy				0.4	0.257
No	64	59 (92.2)	5 (7.8)		
Yes	55	47 (85.5)	8 (14.5)		
Type of RT				0.5	0.387
Short-course IGRT	61	56 (91.8)	5 (8.2)		
Conventional RT	58	50 (86.2)	8 (13.8)		
RT regional nodes				0.6	0.108
No	82	76 (92.7)	6 (7.3)		
Yes	37	30 (81.1)	7 (18.9)		

2P, two-sided *P*-value from Fisher’s exact test; IGRT, image-guided radiotherapy; RT, radiotherapy.

The mean time between breast surgery and the pre-RT physical therapy assessment was 50.5 days (median 38, range 17 to 204). The mean time between pre-RT assessment and start of radiotherapy was 6.2 days (median 6, range 15 to 27), that is, 6 patients had their “pre-RT” assessment delayed to 1, 4, 5, 6, 8, and 15 days after the start of radiotherapy. The mean RT duration was 33 days (median 32, range 18 to 54). The mean time between pre-RT assessment and post-RT assessment was 108.6 days (median 105, range 68 to 235).

The incidence of SA observed at a mean of 50.5 days after surgery but prior to radiotherapy was 10.9% (= 13 of 119). At 1 to 3 months after finishing radiotherapy, which corresponded to a mean time interval of 108.6 days after the pre-RT assessment, SA resolved in 61.5% (= 8 of 13) patients, but persisted in the other 38.5% (= 5 of 13). Moreover, SA appeared after radiotherapy in 1 patient who had no SA at the pre-RT assessment, bringing the post-RT incidence to 5.0% (= 5 + 1 of 119).

Patients’ characteristics are summarized in Table 1. The majority of patients were older than 50 years, with a mean age of 56.6 years (range 32 to 81). Mean pre-RT weight was 68.4 kg (range 42 to 150) and mean height was 1.62 m (range 1.40 to 1.83), resulting in a mean pre-RT BMI of 25.9 kg/m2 (range 17.3 to 51.3). The mean number of lymph nodes examined was 8.2 (range 1 to 35). Taking into account the type of axillary surgery, the mean and range of number of examined nodes were 2.9 (1 to 7) by sentinel node biopsy, and 16.3 (3 to 35) by axillary dissection.

**Figure 3:**
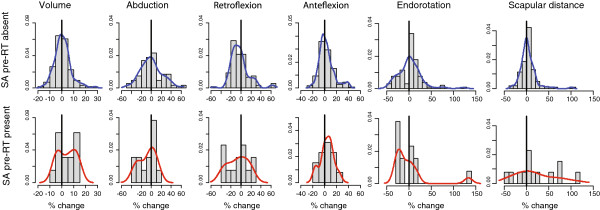
**Histograms of shoulder/arm percent changes, according to scapula alata status.** Curves, continuous density estimates; SA, scapula alata status pre-radiotherapy, Y-axis, relative frequency density.

Factors significantly associated with pre-RT SA were age, body mass index, and axillary dissection (Table 1): pre-RT SA was observed in about 1 of 4 patients younger than 50 years old, in 1 of 5 patients with body mass index less than 25 kg/m2, and in 1 of 5 patients who had axillary dissection. The univariate odds ratios were 4.8, 6.1, and 6.1, respectively. Younger age, lower body mass index, and axillary dissection remained significant factors associated with pre-RT SA in a trimmed multivariate logistic regression (Table 2), as well as in a full model (Appendix 4 in Additional file 1). Figure 3 displays graphically the percentage changes from pre-RT to post-RT, for each of the shoulder/arm assessments, according to patients’ pre-RT SA status. The top row shows outcomes of patient without pre-RT SA, and the bottom row shows the outcomes of patients who presented with pre-RT SA. The histograms show that patients with pre-RT SA presented an increase in arm volume (distribution of bars and density curve shift to >0%), a decrease of abduction (shift to <0%), a decrease of retroflexion (shift to <0%), a small decrease of anteflexion (secondary peak <0%), a decrease of endorotation (shift to <0%), and both increase and decrease of scapular distance (shift to both negative and positive change).

**Table 2:** Multivariate association of patients’ characteristics with pre-radiotherapy scapula alata

	Odds Ratio	95% confidence interval	2*P*
Axillary lymph node dissection vs. sentinel nodes biopsy	10.62	(2.6–57.0)	0.002
Body mass index <25 vs. > = 25	10.53	(2.3–78.7)	0.007
Age at surgery <50 years old vs. > = 50	4.25	(1.1–17.9)	0.037

2*P*, two-sided *P*-value from logisitic regression.

**Figure 4:**
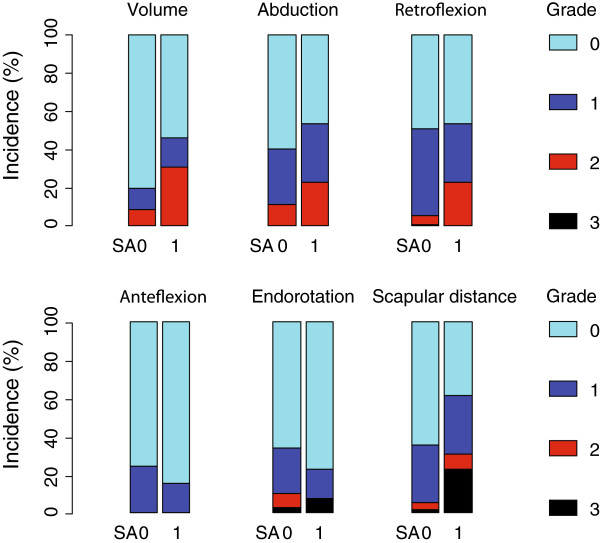
**Toxicity grades according to scapula alata status**. SA, scapula alata pre-radiotherapy status, 0 = absent, 1 = present.

Table 3 summarizes the percentage changes of the measurements that were observed after RT and the corresponding significance tests, according to pre-RT SA status. Measurements were complete, except retroflexion which was imputed in one patient. There were no notable changes of the measurements between pre-RT and post-RT assessments among the non-SA patients, except scapular distance that increased by 3.1%. The overall combined test for non-SA patients was not significant, one-sided *P* = 0.148. Among SA patients, arm volume significantly increased by 4.1%, 1*P* = 0.036, and arm abduction decreased significantly by 8.6%, 1*P* = 0.046 (Table 2). There was also a trend toward decreased retroflexion of 7.0% and increased scapular distance of 19.5% among SA patients. The overall combined test for SA patients reached significance, 1*P* = 0.043. The percentage changes are shown as categorized toxicity grades in Table 4 and Figure 4. Whereas the previous Table 3 evaluated how physical measurements changed over time according to SA status, Table 4 directly compares the changes between the two SA groups of patients. Patients with pre-RT SA presented with more Grade 1 to 3 toxicity by arm volume, abduction, retroflexion, and scapular distance, but comparable endorotation toxicity, and less anteflexion toxicity. Figure 4 displays that patients with pre-RT SA experienced comparatively more frequent toxicities than patients without pre-RT SA, in four out of the six physical assessments (fewer Grade 0, represented as light blue bars), and more frequently with higher grades of toxicities (more Grade 2 and 3, represented as red and black bars).

**Table 3:** Ipsilateral shoulder-arm measurements before and after radiotherapy (RT), according to scapula alata status

	Scapula alata pre-radiotherapy status					
	Absent			Present		
Measurement	Measurement pre-RT	% change post-RT	1*P*	Measurement pre-RT	% change post-RT	1*P*
Volume	1689 (ml)	+0.7%	0.152	1554 (ml)	+4.1%	0.036
Abduction	121 (degrees)	+2.3%	0.818	126 (degrees)	–8.6%	0.046
Retroflexion*	50 (degrees)	–2.5%	0.086	52 (degrees)	–7.0%	0.119
Anteflexion	141 (degrees)	+2.8%	0.987	136 (degrees)	+3.7%	0.871
Endorotation	7 (n vertebrae)	+1.3%	0.304	8 (n vertebrae)	+0.5%	0.485
Scapular distance	14 (cm)	+3.1%	0.037	11 (cm)	+19.5%	0.077
Brown's combined test	–	–	0.148	–	–	0.043

*Retroflexion was imputed in one patient. 1*P*, one-sided *P*-value from paired Student’s t-test.

**Table 4:** Ipsilateral shoulder/arm toxicity according to pre-radiotherapy scapula alata status (SA pre-RT)

	**All**	**SA pre-RT absent**		**SA pre-RT present**		**2*P***
	n	n	(col%)	n	(col%)	
**Volume**						0.013
Grade 0	92	85	(80.2)	7	(53.8)	
Grade 1	14	12	(11.3)	2	(15.4)	
Grade 2	13	9	(8.5)	4	(30.8)	
Grade 3	0	0	(0)	0	(0)	
**Abduction**						0.228
Grade 0	69	63	(59.4)	6	(46.2)	
Grade 1	35	31	(29.2)	4	(30.8)	
Grade 2	15	12	(11.3)	3	(23.1)	
Grade 3	0	0	(0)	0	(0)	
**Retroflexion**						0.312
Grade 0	58	52	(49.1)	6	(46.2)	
Grade 1	52	48	(45.3)	4	(30.8)	
Grade 2	8	5	(4.7)	3	(23.1)	
Grade 3	1	1	(0.9)	0	(0)	
**Anteflexion**						0.463
Grade 0	91	80	(75.5)	11	(84.6)	
Grade 1	28	26	(24.5)	2	(15.4)	
Grade 2	0	0	(0)	0	(0)	
Grade 3	0	0	(0)	0	(0)	
**Endorotation**						0.668
Grade 0	79	69	(65.1)	10	(76.9)	
Grade 1	28	26	(24.5)	2	(15.4)	
Grade 2	8	8	(7.5)	0	(0)	
Grade 3	4	3	(2.8)	1	(7.7)	
**Scapular distance**						0.001
Grade 0	73	68	(64.2)	5	(38.5)	
Grade 1	36	32	(30.2)	4	(30.8)	
Grade 2	5	4	(3.8)	1	(7.7)	
Grade 3	5	2	(1.9)	3	(23.1)	

2*P*, two-sided *P*-value from ordinal trend test.

## Discussion

The present study found a non-negligible incidence of SA in 13 of 119 patients (10.9%) at a mean time interval of 7 weeks (50.5 days) after surgery. Recovery was observed in 8 of the 13 patients 16 weeks (108.6 days) later, in keeping with other observations. Lotze *et al*. reported that serratus anterior palsy occurred in 30% of patients undergoing axillary dissection immediately after surgery, but returned to normal in all patients up to 6 months after the intervention [6]. de Oliveira *et al*. reported that the post-operative incidence of SA was 73.3% immediately after axillary lymphadenectomy, 65.6% after 90 days and 27.7% at the end of follow-up (416 days) [9]. Meininger *et al*. reported that most cases of SA resolved within six to nine months [32]. In Martin and Fish’s review, most cases of isolated serratus anterior palsy resolved with conservative treatment within one to twenty-four months [2].

In line with other authors who compared the SA incidence according to ALND or SNB [4], ALND was one of the most significant risk factor of SA, with SA observed in 10 of 47 (21.3%) ALND patients, as compared with 3 of 72 (4.2%) SNB patients (Table 1). Even though the long thoracic nerve is identified and preserved during axillary dissection [33-36], a higher risk of damage than with sentinel nodes biopsy can be expected to occur. We note that using a logistic regression model that includes age, body mass index, and ALND (Table 2), the expected risk of SA would range from 0.4% in the lowest risk group (older overweight patients treated with SNB), to 63% in the highest risk group (younger leaner patients treated with ALND). This suggests that the variability of SA incidence might be explained, at least in part, by the heterogeneity of populations.

The relationship of lean body weight with increased risk of SA, or conversely the apparent decreased risk of SA with large body weight, is intriguing. We searched the literature on scapular winging of all causes, but found no direct mention of any relationship between SA and weight or BMI. However, in 25 papers that we found reporting pictures of patients, counting multiple photographs of the same patient as only one to avoid duplicated counting, we identified 47 distinct cases: all were lean or average body frame patients, there was no photograph of any overweight case [1,2,10,32,37-57]. The published cases lend support to our observation that weight is inversely related with SA. A tentative explanation is that lean patients might be more at risk of nerve and muscle injury than overweight patients, as there would be less axillary room and fat to move around to spare the long thoracic nerve, and a higher risk of indirect damage by vascular disruption, scarring, or compression against the chest wall. An alternative plausible explanation is that SA is more readily overlooked in overweight patients, in whom positional changes of the scapula would be masked by the overlying adipose tissues. If that is the case, then the true incidence of SA might have been underestimated. We note that in our one patient who had onset of post-RT SA, we found no hint to attribute SA to surgery or to radiotherapy. She was 45 years old, had breast-conserving surgery, sentinel nodes biopsy without ALND, irradiation to the breast without regional node irradiation. But, between the pre-RT assessment and the post-RT assessment, she experienced a weight loss of 10 kg, from a pre-RT weight of 67 kg, her BMI dropped from 24.9 kg/m2 before radiotherapy, to 21.2 kg/m2 thereafter. Incidentally, we found only one case report of SA occurring early after radiotherapy [40]. Our patient would represent the second case so reported to the literature.

Our analyses found that younger age was a significant risk factor for SA. The literature provides scarce and contradictory data regarding age and the incidence of SA after breast cancer surgery. In Pereira *et al*.’s series of patients, the mean age was 60.3 years, but the relationship of SA with age was not investigated [58]. Contrarily to our observation, Ribeiro *et al*. reported in an abstract that age >60 years by logistic regression was associated with an increased SA relative risk of 3.14 [59]. Crude figures were not provided, hence the consistency of Ribeiro *et al*.’s logistic regression with data could not be ascertained, whereas our logistic regression was concordant with our raw data. de Oliveira *et al*. found no significant association of SA with age or any other characteristic [9]. However, in de Oliveira *et al*.’s report, at mean follow-up of 416 days, the relative risk of SA for age >65 vs. age <65 years was 0.53 (95% CI 0.26-1.07), *P* = 0.06, concordantly with our results. There is no obvious explanation why young age would be a risk factor. We can only remark that outside the context of breast cancer, reports of conditions related to scapular winging appear with regard to young and active patients [49,51]. The largest case series of serratus anterior paralysis reported for 197 patients with a mean age of 31.6 years [60]. The literature that we browsed in the discussion about weight was also striking by the preponderance of young patients close to that age.

Regarding the relationship of SA with shoulder/arm morbidity, we encountered two particular issues. One issue is contralateral shoulder/arm morbidity, which we recently found was correlated with ipsilateral morbidity [19]. The other issue was the different measurement scales using different units. We implemented the percentage change of measurement that occurred over time on the ipsilateral limb, therefore avoiding the need to rely on measurements of the contralateral limb, providing the same scale to the measurements, and further allowing links with the common terminology criteria for adverse events [18].

Some discrepancies could be noted in the relationship between SA and shoulder/arm morbidities, such as improved anteflexion and improved endorotation, albeit non-significant (Table 3). Yet, the overall results indicate that SA might be an important early indicator of higher risk of shoulder/arm morbidity. As shown in Table 3 and Figure 3, patients with SA prior to RT (that is, on average seven weeks after surgery) presented more frequently with altered shoulder/arm assessment. Interestingly, the Brown’s combined test which takes into account the correlation between outcomes was significant. This matches the clinical interpretation of shoulder/arm assessments: while each measurement considered separately might show only small alterations, taken all together the measurements might indicate more substantial risk of morbidity, notably lymphedema or loss of motion (Table 4 and Figure 4).

We are aware of the limitations of the present study. No physical assessment was done prior to surgery, precluding the possibility of analyzing the impact of pre-existing morbidities. The number of patients was small, which did not allow comprehensive analyses, modeling gave results difficult to interpret (Appendix 6 in Additional file 1), therefore limiting the scope of the present study to a descriptive stance. The follow-up was short. Though SA appeared as a predictor of early shoulder/arm toxicities, its value as a predictor of long-term toxicities remains unknown. We did not assess compliance of patients with preventive physical therapy. We did not assess the reproducibility of measurements. It has been argued there is no consistent evidence that any examination procedure used in shoulder assessments has acceptable levels of reliability [61]. Contrariwise, assessment of scapular positioning and winging has been reported to be reliable [62,63]. In order to evaluate inter-observer variability, the present study could have benefited from repeated assessment by different observers. This was not built into the trial’s design in view of the trial’s time constraints and examinations that patients underwent. Until the present study, we had no a priori reason to give precedence to SA assessment. The study could also have benefited from advanced scapular motion tracking and from electromyographic confirmation of serratus palsy [64]. But, for the same reason that multi-observer assessments were not done, there was no a priori indication to perform motion tracking or electromyography.

The strengths of the study are its prospective nature, the patients were consistently evaluated clinically by the same team within the same institution throughout the study duration. Good internal consistency of measurements done by the same observer could be expected [13,62]. The physical therapy assessment was blinded to patients’ randomization allocation. Furthermore, the assessors were not involved in the physical therapeutic management of the patients. We have mentioned as a limitation that compliance was not assessed. Yet, this concurred to strengthen the study against bias that could have resulted from knowing patients’ treatments. We believe that the results are robust and warrant further investigations.

## Conclusion

In this study, we analyzed the change of SA incidence after post-surgery radiotherapy for breast cancer and the physical functioning factors related with SA. The results confirm the previously known association of SA with ALND. We found an inverse association of SA with age and body weight, not previously reported in the literature, the latter suggesting that SA might have been underestimated. Post-surgery SA appeared to recover in a majority of patients at 15 weeks of follow-up. However, we also found that despite the recovery, SA portended an increased risk of loss of shoulder-arm mobility.

We argue that scapular winging is not an innocuous sign, that it should be actively evaluated in order to identify patients who might be most at need of close physical therapy management.

## Abbreviations

ALND: Axillary lymph node dissection; IGRT: Image-guided radiotherapy; RT: Radiotherapy; SA: Scapula alata; SNB: Sentinel nodes biopsy.

## Competing interests

The authors declare that they have no competing interests. The first author (NA) is a bursary of the IWT, Belgian Agency for Innovation by Science and Technology, http://www.iwt.be/. The trial was funded by grant SCIE2006-30 from the Stichting tegen Kanker, Belgian Foundation against Cancer, http://www.kanker.be/. The Radiotherapy Department of the University Hospital, Brussels had a research agreement with TomoTherapy Inc. (Madison, WI, USA) and Orfit Industries (Wijnegem, Belgium). None of the funding agencies were involved in the study design; in the collection, analysis and interpretation of data; in the writing of the manuscript; or in the decision to submit the manuscript for publication.

## Authors’ contributions

NA was the trial's co-investigator, designed the study, collected and analysed the data, and drafted the manuscript. MDR was the trial's director, edited and critically reviewed the manuscript. PL, MVo, and GS edited and critically reviewed the manuscript. HVP was the trial's co-investigator, collected the data, and drafted the manuscript. MVa collected the data, ensured patients’ follow-up, and edited the manuscript. GM collected data, evaluated patients, edited and critically reviewed the manuscript. HV did the data management, and drafted the manuscript. JL contributed to patients’ follow-up, edited and reviewed the manuscript. SP contributed to data collection and to manuscript writing. VVH was the trial's principal investigator, provided the study concept, analysed the data analysis, and wrote the manuscript. All authors read and approved the final manuscript.

## Supplementary Material

Additional file 1 Appendix 1. Computing volume from circumference measurements for TomoBreast patients: Appendix 2. A list of variables used for imputation of missing data; Appendix 3. The relationships between scapula alata and characteristics; Appendix 4. Logistic regression, all variables without selection; Appendix 5. Figure of shoulder/arm percentage changes from pre- to post-radiotherapy, according to scapula alata status; Appendix 6: Post-radiotherapy outcomes (percentage change of shoulder/arm measurement between pre- and post-RT) and linear predictors. Click here for file

Additional file 2 File format PDF. Brown.combined.Pvalues. Click here for file

## References

[B1] Vanderstraeten J Scapula alata Rev Med Gen 2010 269 32 33

[B2] Martin RM Fish DE Scapular winging: anatomical review, diagnosis, and treatments Curr Rev Musculoskelet Med 2008 1 1 11 10.1007/s12178-007-9000-5 19468892 PMC2684151

[B3] Kauppila LI Vastamaki M Iatrogenic serratus anterior paralysis. Long-term outcome in 26 patients Chest 1996 109 31 34 10.1378/chest.109.1.31 8549212

[B4] Paim CR de Paula Lima ED Fu MR de Paula LA Cassali GD Post lymphadenectomy complications and quality of life among breast cancer patients in Brazil Cancer Nurs 2008 31 302 309 10.1097/01.NCC.0000305747.49205.b1 18600117

[B5] Velpeau AALM Traite d’anatomie chirurgicale ou anatomie des regions, consideree dans ses rapports avec la chirurgie 1825 Paris, France: Crevot PMC5948463 30332278

[B6] Lotze MT Duncan MA Gerber LH Woltering EA Rosenberg SA Early versus delayed shoulder motion following axillary dissection: a randomized prospective study Ann Surg 1981 193 288 295 10.1097/00000658-198103000-00007 7011221 PMC1345064

[B7] Roses DF Brooks AD Harris MN Shapiro RL Mitnick J Complications of level I and II axillary dissection in the treatment of carcinoma of the breast Ann Surg 1999 230 194 201 10.1097/00000658-199908000-00009 10450733 PMC1420861

[B8] Saied GM Kamel RM Dessouki NR The effect of mastectomy and radiotherapy for breast carcinoma on soft tissues of the shoulder and its joint mobility among Egyptian patients Tanzan Health Res Bull 2007 9 121 125 17722415 10.4314/thrb.v9i2.14314

[B9] de Oliveira JF Bezerra T Ribeiro ACP Dias RA Abrahao F Silva JG Bergmann A Incidence and risk factors of winged scapula after axillary lymph node dissection in breast cancer surgery Appl Cancer Res 2009 29 69 73

[B10] de Sousa Mastrella A Freitas-Junior R Paulinelli RR Soares LR Escápula alada pós-linfadenectomia no tratamento do câncer de mama Rev Bras Cancerologia 2009 55 397 404

[B11] Efron B Forcing a sequential experiment to be balanced Biometrika 1971 58 403 417 10.1093/biomet/58.3.403

[B12] Karges JR Mark BE Stikeleather SJ Worrell TW Concurrent validity of upper-extremity volume estimates: comparison of calculated volume derived from girth measurements and water displacement volume Phys Ther 2003 83 134 145 12564949

[B13] Nijs J Roussel N Vermeulen K Souvereyns G Scapular positioning in patients with shoulder pain: a study examining the reliability and clinical importance of 3 clinical tests Arch Phys Med Rehabil 2005 86 1349 1355 10.1016/j.apmr.2005.03.021 16003663

[B14] Kibler WB Role of the scapula in the overhead throwing motion Contemp Orthop 1991 22 525 532

[B15] Kibler WB The role of the scapula in athletic shoulder function Am J Sports Med 1998 26 325 337 9548131 10.1177/03635465980260022801

[B16] Mottram SL Dynamic stability of the scapula Man Ther 1997 2 123 131 10.1054/math.1997.0292 11440525

[B17] de Groot JH The scapulo-humeral rhythm: effects of 2-D roentgen projection Clin Biomech (Bristol, Avon) 1999 14 63 68 10.1016/S0268-0033(98)00027-8 10619091

[B18] National Cancer Institute Common Terminology Criteria for Adverse Events (CTCAE). Version 4.0. NIH Publication No. 09-5410 2010 Revised June 2010

[B19] Adriaenssens N Vinh-Hung V Miedema G Versmessen H Lamote J Vanhoeij M Lievens P Van Parijs H Storme G Voordeckers M Early contralateral shoulder-arm morbidity in breast cancer patients enrolled in a randomized trial of post-surgery radiation therapy Breast Cancer 2012 in press 10.4137/BCBCR.S9362 PMC3418149 22904635

[B20] Riddle DL Rothstein JM Lamb RL Goniometric reliability in a clinical setting. Shoulder measurements Phys Ther 1987 67 668 673 3575423 10.1093/ptj/67.5.668

[B21] Barnes CJ Van Steyn SJ Fischer RA The effects of age, sex, and shoulder dominance on range of motion of the shoulder J Shoulder Elbow Surg 2001 10 242 246 10.1067/mse.2001.115270 11408905

[B22] Conte AL Marques AP Casarotto RA Amado-Joao SM Handedness influences passive shoulder range of motion in nonathlete adult women J Manipulative Physiol Ther 2009 32 149 153 10.1016/j.jmpt.2008.12.006 19243727

[B23] Roy JS MacDermid JC Boyd KU Faber KJ Drosdowech D Athwal GS Rotational strength, range of motion, and function in people with unaffected shoulders from various stages of life Sports Med Arthrosc Rehabil Ther Technol 2009 1 4 10.1186/1758-2555-1-4 19284527 PMC2663552

[B24] Mullaney MJ McHugh MP Johnson CP Tyler TF Reliability of shoulder range of motion comparing a goniometer to a digital level Physiother Theory Pract 2010 26 327 333 10.3109/09593980903094230 20557263

[B25] Van Hoof T Vangestel C Shacklock M Kerckaert I D’Herde K Asymmetry of the ULNT1 elbow extension range-of-motion in a healthy population: Consequences for clinical practice and research Phys Ther Sport 2012 in press 10.1016/j.ptsp.2011.09.003 22814447

[B26] Agresti A Categorical data analysis 2002 2 Hoboken NJ: Wiley

[B27] Armitage P Berry G Matthews JNS Statistical Methods in Medical Research 2002 Malden, MA: Blackwell Science Reprinted 2007. ISBN Fourth

[B28] Brown MB A method for combining non-independent, one-sided tests of significance Biometrics 1975 31 987 992 10.2307/2529826

[B29] R Development Core Team R: A language and environment for statistical computing R Foundation for Statistical Computing: Vienna, Austria http://www.R-project.org/

[B30] van Buuren S Multiple imputation of discrete and continuous data by fully conditional specification Stat Methods Med Res 2007 16 219 242 10.1177/0962280206074463 17621469

[B31] Venables WN Ripley BD Modern Applied Statistics with S 2002 4 New York: Springer-Verlag

[B32] Meininger AK Figuerres BF Goldberg BA Scapular winging: an update J Am Acad Orthop Surg 2011 19 453 462 21807913 10.5435/00124635-201108000-00001

[B33] Auchincloss H Significance of location and number of axillary metastases in carcinoma of the breast: a justification for a conservative operation Ann Surg 1963 158 37 46 10.1097/00000658-196307000-00008 14042633 PMC1408351

[B34] Petrek JA Blackwood MM Axillary dissection: current practice and technique Curr Probl Surg 1995 32 257 323 10.1016/S0011-3840(05)80015-2 7705102

[B35] Martin JK Axillary dissection Oper Tech Gen Surg 2000 2 152 160 10.1053/otgn.2000.7064

[B36] Mostafa A Mokbel K Engledow A Leris AC Choy C Wells C Carpenter R Is dissection of the internerve tissue during axillary lymphadenectomy for breast cancer necessary? Eur J Surg Oncol 2000 26 153 154 10.1053/ejso.1999.0760 10744934

[B37] Overpeck DO Ghormley RK Paralysis of the serratus magnus muscle, caused by lesions of the long thoracic nerve JAMA 1940 114 1994 1996

[B38] Ilfeld FW Holder HG Winged scapula: case occurring in soldier from knapsack JAMA 1942 120 448 449 10.1001/jama.1942.82830410004008a

[B39] Duncan MA Lotze MT Gerber LH Rosenberg SA Incidence, recovery, and management of serratus anterior muscle palsy after axillary node dissection Phys Ther 1983 63 1243 1247 6878433 10.1093/ptj/63.8.1243

[B40] Pugliese GN Green RF Antonacci A Radiation-induced long thoracic nerve palsy Cancer 1987 60 1247 1248 10.1002/1097-0142(19870915)60:6<1247::AID-CNCR2820600615>3.0.CO;2-R 3040210

[B41] Post M Pectoralis major transfer for winging of the scapula J Shoulder Elbow Surg 1995 4 1 9 10.1016/S1058-2746(10)80001-1 7874558

[B42] Watson CJ Schenkman M Physical therapy management of isolated serratus anterior muscle paralysis Phys Ther 1995 75 194 202 7870751 10.1093/ptj/75.3.194

[B43] Kibler WB Uhl TL Maddux JW Brooks PV Zeller B McMullen J Qualitative clinical evaluation of scapular dysfunction: a reliability study J Shoulder Elbow Surg 2002 11 550 556 10.1067/mse.2002.126766 12469078

[B44] Wiater JM Flatow EL Long thoracic nerve injury Clin Orthop Relat Res 1999 368 17 27 10613149

[B45] Dumontier C Soubeyran M Lascar T Laulan J Compression du nerf thoracicus longus (Nerf de Charles-Bell) Chir Main 2004 23 S63 S76

[B46] Sherman SC O’Connor M An unusual cause of shoulder pain: Winged scapula J Emerg Med 2005 28 329 331 10.1016/j.jemermed.2004.08.022 15769579

[B47] Lee SG Kim JH Lee SY Choi IS Moon ES Winged scapula caused by rhomboideus and trapezius muscles rupture associated with repetitive minor trauma: a case report J Korean Med Sci 2006 21 581 584 10.3346/jkms.2006.21.3.581 16778411 PMC2729973

[B48] Vinson EN Clinical images: scapular winging Arthritis Rheum 2006 54 4027 10.1002/art.22274 17133539

[B49] Daubinet G Graveleau N Rousseau D L’epaule du sportif. The athletes shoulder Rev Rhum 2007 74 581 586 10.1016/j.rhum.2007.04.003

[B50] Nath RK Melcher SE Rapid recovery of serratus anterior muscle function after microneurolysis of long thoracic nerve injury J Brachial Plex Peripher Nerve Inj 2007 2 4 10.1186/1749-7221-2-4 17291339 PMC1802864

[B51] Noel E Les syndromes canalaires de l’epaule. Nerve entrapment of the shoulder Rev Rhum 2007 74 339 343 10.1016/j.rhum.2007.02.019

[B52] Galano GJ Bigliani LU Ahmad CS Levine WN Surgical treatment of winged scapula Clin Orthop Relat Res 2008 466 652 660 10.1007/s11999-007-0086-2 18196359 PMC2505206

[B53] Aksoy IA Schrader SL Ali MS Borovansky JA Ross MA Spinal accessory neuropathy associated with deep tissue massage: a case report Arch Phys Med Rehabil 2009 90 1969 1972 10.1016/j.apmr.2009.06.015 19887226

[B54] Cerqueira WA Barbosa LA Bergmann A Proposta de conduta fisioterapêutica para o atendimento ambulatorial nas pacientes com escápula alada após linfadenectomia axilar Rev Bras Cancerologia 2009 55 115 120

[B55] McClure P Tate AR Kareha S Irwin D Zlupko E A clinical method for identifying scapular dyskinesis, part 1: reliability J Athl Train 2009 44 160 164 10.4085/1062-6050-44.2.160 19295960 PMC2657031

[B56] Sivan M Hassan A Images in emergency medicine. Winged scapula as the presenting symptom of Guillain-Barre syndrome Emerg Med J 2009 26 790 10.1136/emj.2008.066613 19850802

[B57] Blum A Lecocq S Louis M Wassel J Moisei A Teixeira P The nerves around the shoulder Eur J Radiol 2011 [epub ahead of print] 10.1016/j.ejrad.2011.04.033 21546184

[B58] Pereira TB Bergmann A Ribeiro AC Da Silva JG Dias R Ribeiro MJ Thuler LC Myoeletric activity pattern of scapular muscles after axillary lymphadenectomy in breast cancer Rev Bras Ginecol Obstet 2009 31 224 229 19669029 10.1590/s0100-72032009000500004

[B59] Ribeiro A Bergmann A Bezerra T Silva M Silva J Ribeiro M Dias R Incidência de escápula alada no pós-operatório de linfadenectomia axilar [abstract] Rev Bras Cancerologia 2007 53 491

[B60] Vastamaki M Kauppila LI Etiologic factors in isolated paralysis of the serratus anterior muscle: a report of 197 cases J Shoulder Elbow Surg 1993 2 240 243 10.1016/S1058-2746(09)80082-7 22959502

[B61] May S Chance-Larsen K Littlewood C Lomas D Saad M Reliability of physical examination tests used in the assessment of patients with shoulder problems: a systematic review Physiotherapy 2010 96 179 190 10.1016/j.physio.2009.12.002 20674649

[B62] Nijs J Roussel N Struyf F Mottram S Meeusen R Clinical assessment of scapular positioning in patients with shoulder pain: state of the art J Manipulative Physiol Ther 2007 30 69 75 10.1016/j.jmpt.2006.11.012 17224359

[B63] Struyf F Nijs J De CK Giunta M Mottram S Meeusen R Clinical assessment of scapular positioning in musicians: an intertester reliability study J Athl Train 2009 44 519 526 10.4085/1062-6050-44.5.519 19771291 PMC2742462

[B64] Struyf F Nijs J Horsten S Mottram S Truijen S Meeusen R Scapular positioning and motor control in children and adults: a laboratory study using clinical measures Man Ther 2011 16 155 160 10.1016/j.math.2010.09.002 20951074

