# Supplementary material for: Scapula alata in early breast cancer patients enrolled in a randomized clinical trial of post-surgery short-course image-guided radiotherapy
Source: World J Surg Oncol. 2012 May 16;10:86. doi: 10.1186/1477-7819-10-86 (PMC3488523; doi:10.1186/1477-7819-10-86)
Supplement: Additional file 1 — Appendix 1. Computing volume from circumference measurements for TomoBreast patients: Appendix 2. A list of variables used for imputation of missing data; Appendix 3. The relationships between scapula alata and characteristics; Appendix 4. Logistic regression, all variables without selection; Appendix 5. Figure of shoulder/arm percentage changes from pre- to post-radiotherapy, according to scapula alata status; Appendix 6: Post-radiotherapy outcomes (percentage change of shoulder/arm measurement between pre- and post-RT) and linear predictors. [file 1477-7819-10-86-S1.doc]

# Additional File 1

## Appendix 1. Computing volume from circumference measurements for TomoBreast patients

| Level where circumference is taken | Recorded example | Volume of segment by frustum method | Volume of segment by cylinder method |
| --- | --- | --- | --- |
| 15cm above elbow | 26.0 cm |  |  |
|  |  | 269.0 cm^3^ | 269.0 cm^3^ |
| 10cm above elbow | 26.0 cm |  |  |
|  |  | 761.3 cm^3^ | 761.0 cm^3^ |
| 5cm below olecranon | 24.5 cm |  |  |
|  |  | 229.2 cm^3^ | 229.2 cm^3^ |
| 10 cm below olecranon | 23.5 cm |  |  |
|  |  | 192.9 cm^3^ | 192.6 cm^3^ |
| 15 cm below olecranon | 20.5 cm |  |  |
|  |  |  |  |
| Total |  | 1452.3 cm^3^ | 1451.8 cm^3^ |

**Frustum method**

The arm is modeled as successive cones.

Volume of a segment of cone between two discs *c_1_* and *c_2_* separated by height *h*

= *h* x (*c_1_*^2^ + *c_1_* x *c_2_* + *c_2_*^2^) / (12 x PI)

**Cylinder method**

The arm is modeled as successive cylinders.

Volume of a segment of cylinder between two discs *c1* and *c2* separated by height *h*

= *h* x [(*c_1_* + *c_2_*)/2]^2^ / (4 x PI)

The two methods give different results. In the above example, volume by frustum method = 1452.3 cm^3^, by cylinder method = 1451.8 cm^3^, the mean computed volume from the two methods = 1452.0 cm^3^.

Reference:

Karges JR, Mark BE, Stikeleather SJ, Worrell TW. Concurrent validity of upper-extremity volume estimates: comparison of calculated volume derived from girth measurements and water displacement volume. Phys Ther 2003, 83:134-145.

## Appendix 2: List of variables used for imputation of missing data

Labels naming elements:

T0 = pre-RT assessment.

T1 = post-RT assessment.

O = ipsilateral arm.

NO = contralateral arm.

armSymptoms = responses to structured EORTC BR-23 questionnaire (included for imputation but not analyzed in the present study).

breastSymptoms = responses to structured EORTC BR-23 questionnaire (included for imputation but not analyzed in the present study).

subj.edema = unstructured presence or absence of arm symptoms.

| **Label** | **Remark** |
| --- | --- |
| "ktime.T0" | Time of pre-RT assessement (T0) |
| "ktime.T1" | Time of pre-RT assessement (T1) |
| "height" | Patient's height |
| "SideIsDominant" | Operated side is side of dominant arm |
| "armSymptoms.T0" | EORTC BR-23 arm symptoms at T0 |
| "armSymptoms.T1" |  |
| "breastSymptoms.T0" | EORTC BR-23 breast symptoms at T0 |
| "breastSymptoms.T1" |  |
| "weight.T0" |  |
| "weight.T1" |  |
| "volume.O.T0" |  |
| "volume.O.T1" |  |
| "volume.NO.T0" |  |
| "volume.NO.T1" |  |
| "subj.edema.T0" |  |
| "subj.edema.T1" |  |
| "anteflex.O.T0" |  |
| "anteflex.O.T1" |  |
| "anteflex.NO.T0" |  |
| "anteflex.NO.T1" |  |
| "retroflex.O.T0" |  |
| "retroflex.O.T1" |  |
| "retroflex.NO.T0" |  |
| "retroflex.NO.T1" |  |
| "abduction.O.T0" |  |
| "abduction.O.T1" |  |
| "abduction.NO.T0" |  |
| "abduction.NO.T1" |  |
| "scap.dist.O.T0" |  |
| "scap.dist.O.T1" |  |
| "scap.dist.NO.T0" |  |
| "scap.dist.NO.T1" |  |
| "scap.wing.T0" |  |
| "scap.wing.T1" |  |
| "endorot.O.T0" |  |
| "endorot.O.T1" |  |
| "endorot.NO.T0" |  |
| "endorot.NO.T1" |  |
| "DoseGy" | Radiotherapy dose given |
| "tomo" | Randomization allocation group (intent to treat) |
| "BCS" | Breast conserving surgery indicator |
| "ALND" | Axillary dissection indicator |
| "Chemo" | Chemotherapy |
| "Chemoschedule" | Chemotherapy schedule |
| "nodalRT" | Radiation of nodal areas |
| "HT" | Hormone therapy |
| "AI" | Aromatase inhibitor |
| "AgeAtSurg" | Age at surgery |

## Appendix 3: Relationships between scapula alata and characteristics

### Logistic regression, stepwise selection of variables

fit0 <- glm(scap.wing.T0~1,data=mc.im1,family=binomial)

summary(fit1 <- stepAIC(fit0,scope=list(upper=~I(AgeAtSurg<50) + I(weight.T0<70)

+ I(height>=1.60) + I(BMI.T0<25) + subj.edema.T0

+ SideIsDominant + BCS + ALND + Chemo + tomo + nodalRT,

lower=~1), direction="both",trace=0))

Result:

| Coefficients: | |  |  |  |
| --- | --- | --- | --- | --- |
|  | Estimate | Std.Error | zvalue | Pr(>|z|) |
| (Intercept) | -5.638 | 1.120 | -5.035 | 0.000 |
| ALND | 2.363 | 0.767 | 3.080 | 0.002 |
| I(BMI.T0<25)TRUE | 2.355 | 0.868 | 2.711 | 0.007 |
| I(AgeAtSurg<50)TRUE | 1.447 | 0.693 | 2.089 | 0.037 |

# adjusted odds ratio with 95% CI

cbind(oddsratio=exp(fit1$coef), exp(confint(fit1)) )

|  | oddsratio | 2.50% | 97.50% |
| --- | --- | --- | --- |
| (Intercept) | 0.00 | 0.00 | 0.02 |
| ALND | 10.62 | 2.62 | 57.05 |
| I(BMI.T0<25)TRUE | 10.53 | 2.28 | 78.71 |
| I(AgeAtSurg<50)TRUE | 4.25 | 1.12 | 17.94 |

## Appendix 4: Logistic regression, all variables without selection

summary(fit2 <- glm(scap.wing.T0~I(AgeAtSurg<50) + I(weight.T0<70)

+ I(height>=1.60) + I(BMI.T0<25) + subj.edema.T0

+ SideIsDominant + BCS + ALND + Chemo + tomo + nodalRT,

data=mc.im1,family=binomial))

Result:

| Coefficients: | |  |  |  |
| --- | --- | --- | --- | --- |
|  | Estimate | Std.Error | zvalue | Pr(>|z|) |
| (Intercept) | -5.516 | 1.657 | -3.329 | 0.001 |
| I(AgeAtSurg<50)TRUE | 1.987 | 0.828 | 2.401 | 0.016 |
| I(weight.T0<70)TRUE | -0.242 | 1.413 | -0.171 | 0.864 |
| I(height>=1.6)TRUE | -0.995 | 0.866 | -1.148 | 0.251 |
| I(BMI.T0<25)TRUE | 2.843 | 1.369 | 2.077 | 0.038 |
| subj.edema.T0 | -1.016 | 1.021 | -0.995 | 0.320 |
| SideIsDominant | 0.472 | 0.861 | 0.548 | 0.584 |
| BCS | 0.431 | 0.845 | 0.510 | 0.610 |
| ALND | 2.489 | 1.136 | 2.190 | 0.029 |
| Chemo | -0.679 | 0.893 | -0.760 | 0.447 |
| tomo | -0.268 | 0.772 | -0.347 | 0.729 |
| nodalRT | 0.733 | 1.042 | 0.704 | 0.482 |

cbind(oddsratio=exp(fit2$coef), exp(confint(fit2)) )

|  | oddsratio | 2.50% | 97.50% |
| --- | --- | --- | --- |
| (Intercept) | 0.004 | 0.000 | 0.068 |
| I(AgeAtSurg<50)TRUE | 7.292 | 1.568 | 43.749 |
| I(weight.T0<70)TRUE | 0.785 | 0.046 | 13.897 |
| I(height>=1.6)TRUE | 0.370 | 0.062 | 2.031 |
| I(BMI.T0<25)TRUE | 17.173 | 1.558 | 365.110 |
| subj.edema.T0 | 0.362 | 0.040 | 2.406 |
| SideIsDominant | 1.603 | 0.282 | 9.010 |
| BCS | 1.538 | 0.294 | 8.685 |
| ALND | 12.045 | 1.474 | 140.774 |
| Chemo | 0.507 | 0.080 | 2.904 |
| tomo | 0.765 | 0.159 | 3.513 |
| nodalRT | 2.081 | 0.285 | 18.436 |

## Appendix 5: Figure of shoulder/arm percent changes from pre- to post-radiotherapy, according to scapula alata status

SA: Scapula alata status pre-radiotherapy, 0 = not present, 1 = present.

Box convention: median (thick line); lower and upper hinges close to first and third quartile. Lower and upper whiskers extend to the most extreme data point which is no more than 1.5 times the length of the box away from the box.

## Appendix 6: Post-radiotherapy outcomes (percent change of shoulder/arm measurement between pre- and post-RT) and linear predictors

| **Outcome (*)** | **Linear predictors** | **Linear coefficient** | **2P** | **AIC** |
| --- | --- | --- | --- | --- |
| Volume | - Body weight increase (per kg) | 0.5 | <0.001 | -11.4 |
|  | - Axillary dissection (vs. sentinel nodes biopsy) | 3.8 | 0.002 | -7.7 |
|  | - Type of RT (IGRT vs. conventional RT) | -3.5 | 0.004 | -6.4 |
| Abduction | - Arm symptoms pre-RT (yes vs. no) | 15.0 | 0.012 | -4.6 |
|  | - Type of RT (IGRT vs. conventional RT) | 8.4 | 0.066 | -1.5 |
|  | - Regional nodes irradiation (yes vs. no) | -8.4 | 0.106 | -0.7 |
|  | - Scapula alata (yes vs. no) | -10.4 | 0.158 | -0.1 |
| Retroflexion | - – | – | – | – |
| Anteflexion | - Arm symptoms pre-RT (yes vs. no) | 8.1 | 0.003 | -7.0 |
|  | - Breast conserving surgery | -5.9 | 0.009 | -4.9 |
| Endorotation | - Arm symptoms pre-RT (yes vs. no) | -17.5 | 0.008 | -5.3 |
|  | - Age at surgery | 0.4 | 0.076 | -1.3 |
|  | - Operation side is dominant arm | 7.7 | 0.140 | -0.3 |
| Scapular distance | - Scapula alata (yes vs. no) | 15.2 | 0.021 | -3.5 |
|  | - Operation side is dominant arm | 7.6 | 0.065 | -1.5 |

(*) Outcome defined as percent change of measurement on the ipsilateral limb between pre- and post-radiotherapy assessment.

AIC: Akaike information criteria, more negative value indicates better predictor. RT: radiotherapy. IGRT: image guided radiotherapy. Retroflexion: no informative predictor found.

Linear coefficient: indicates the expected post-radiotherapy % increase or decrease of an outcome that is attributable to a predictor.
