# Supplementary material for: Scapula alata in early breast cancer patients enrolled in a randomized clinical trial of post-surgery short-course image-guided radiotherapy
Source: World J Surg Oncol. 2012 May 16;10:86. doi: 10.1186/1477-7819-10-86 (PMC3488523; doi:10.1186/1477-7819-10-86)
Supplement: Additional file 2 — File format PDF. Brown.combined.Pvalues. [file 1477-7819-10-86-S2.pdf]

```
#####################
#
# Fisher 1948 combined test of significance for independent tests
# Mosteller F, Fisher RA. Combining independent tests of significance.
# The American Statistician, Vol. 2, No. 5 (Oct., 1948), pp. 30-31.
# Brown MB. A Method for Combining Non-Independent,
# One-Sided Tests of Significance.
# Biometrics, Vol. 31, No. 4 (Dec., 1975), pp. 987-992.
# R 2.14.1's implementation Vincent Vinh-Hung
# March 12, 2012
# Manuscript WJSO 'scapula alata'
#
#####################

# data frame of shoulder/arm measurements
y <- mc.im1[,c("volume.O.rc1", "abduction.O.rc1"
        , "retroflex.O.rc1", "anteflex.O.rc1"
        , "endorot.O.rc1", "scap.dist.O.rc1")]
y[,2] <- -y[,2] # see manuscript's note on morbidity sign convention
y[,3] <- -y[,3] #
y[,4] <- -y[,4] #
# pre-RT scapula alata indicator, 0=no, 1=yes
x <- mc.im1$scap.wing.T0

# Fisher's combined X2

fX2 <- c(-2*sum(log(sapply(1:ncol(y), function(i)
        t.test(y[,i][x==0],mu=0,alternative="greater")$p.value ))),
        -2*sum(log(sapply(1:ncol(y), function(i)
        t.test(y[,i][x==1],mu=0,alternative="greater")$p.value ))) )
1-pchisq(fX2,ncol(y)*2)

# Brown 1975

# E(X^2) = 2 * k, where k = number of tests
# s^2(X^2) = 4 * k + 2 * sumsum i<j cov(-2log pi , -2 log pj)
# where cov(-2log pi , -2 log pj) =
# r * (3.25 + .75 * r) if 0<=r<=1
# r * (3.27 + .71 * r) if -.5 <= r <= 0
# or get cov from Brown's table
# c(0,.334,.681,1.044,1.421,1.812,2.219,2.641,3.079,3.531,4.000)
# c(0,-.320,-.625,-.916,-1.194,-1.458,-1.709,-1.946,-2.17,-2.382,-2.590)
# last value -2.590 was not in Brown's original paper,
# added by VVH to simplify interpolation using R's function approx
tmp <-
c(-2.59,-2.382,-2.17,-1.946,-1.709,-1.458,-1.194,-.916,-.625,-.320,
        0,.334,.681,1.044,1.421,1.812,2.219,2.641,3.079,3.531,4)
s2X2 <- 4 * ncol(y) + 2 * sum ( approx( seq(-1,1,.1),tmp,
        xout=cor(y)[which( as.vector(lower.tri(cor(y))) )] )$y )
# newdf "f" = 2 * (2 * k)^2 /s2X2
# X2 ccorrection "c" = s2X2/(2*2*k)
1-pchisq(fX2/(s2X2/(2*2*ncol(y))), 2 * (2 * ncol(y))^2 /s2X2)
```
